# A review of community-acquired methicillin-resistant *Staphylococcus aureus* for primary care physicians

**DOI:** 10.4103/1319-1683.74320

**Published:** 2010

**Authors:** Huda A. Bukharie

**Affiliations:** *Department of Internal Medicine, Infectious Disease Unit, King Fahd Hospital of the University, Al-Khobar, Kingdom of Saudi Arabia*

**Keywords:** Clindamycin, methicillin, Saudi Arabia, *Staphylococcus aureus*

## Abstract

Community-acquired methicillin-resistant *Staphylococcus aureus* (CA-MRSA) infections among young people without healthcare-associated risk factors have emerged during the past decade. Reported prevalence rates of CA-MRSA vary widely among studies, largely because of the different definitions employed and different settings in which the studies have been performed. Although the majority of CA-MRSA infections are mild skin and soft tissue infections, severe life-threatening cases have been reported. CA-MRSA infections have mostly been associated with staphylococcal strains bearing the staphylococcal cassette chromosome mec type IV element and Panton-Valentine leukocidin genes. These strains are more frequently susceptible to a variety of non-beta-lactam antibiotics. Clinicians must be aware of the wide spectrum of disease caused by CA-MRSA. Continued emergence of MRSA in the community is a public health problem, and therefore warrants increased vigilance in the diagnosis and management of suspected and confirmed staphylococcal infections.

## INTRODUCTION

Methicillin-resistant *Staphylococcus aureus* (MRSA) is an important cause of nosocomial infections worldwide. However, the epidemiology of MRSA is changing as the isolation of MRSA is no longer limited to hospitalized patients or persons with predisposing risk factors.[1] Outbreaks of community-associated MRSA (CA-MRSA) have been reported worldwide in diverse community populations.[2–4] CA-MRSA strains are now recognized as distinct clonal entities that differ from traditional hospital-acquired MRSA (HA-MRSA) strains. By definition, both CA-MRSA and HA-MRSA are resistant to methicillin (and all beta-lactam antibiotics), but important differences exist in epidemiology, microbiologic characteristics, clinical aspects of infection, and management strategies between the two.

### Definition

Reported prevalence rates of CA-MRSA vary widely among studies, in part because of the use of different definitions to distinguish between CA-MRSA and HA-MRSA, but also because of the different settings in which studies have been performed. It should be noted that relatively few studies have been conducted among randomly selected healthy members of the community. Most studies have been based on hospitalized patients or patients upon admission to the hospital, which has probably resulted in an overestimation of the ‘true’ prevalence of CA-MRSA.

No consensus definition for CA-MRSA exists. In February 2005, the Centers for Infectious Disease Control and Prevention defined CA-MRSA infection as identification of MRSA in a patient with signs and symptoms of infection, either in the outpatient setting or within 48 hours after admission to a hospital, with no history of MRSA infection or colonization, no history of admission to a hospital or a nursing home during the previous year, and no history of dialysis, surgery, permanent indwelling catheters, or medical devices that pass through the skin to the body.[5]

### Microbiology and genetics

CA-MRSA is distinct from HA-MRSA both genetically and phenotypically. Methicillin resistance, signifying resistance to all beta-lactam antibiotics, is mediated by the mecA gene which codes for the penicillin-binding protein PBP2A. The mecA gene is located on a genetic island called the staphylococcal cassette chromosome mec (SCCmec), and differences in SCCmec are used to categorize MRSA. HA-MRSA strains carry SCCmec types I to III, whereas CA-MRSA strains carry SCCmec IV and the more recently isolated SCCmec V; HA-MRSA tends to be multiresistant, whereas CA-MRSA tends to be susceptible to narrow-spectrum non-beta-lactams such as clindamycin, trimethoprim-sulfamethoxazole (TMP-SMX), and tetracyclines.[6,7]

Another distinguishing genetic feature of CA-MRSA is that a high percentage of strains carry genes for Panton-Valentine leukocidin (PVL), an exotoxin that is lethal to leukocytes. Genes for PVL are largely absent from HA-MRSA strains. PVL, perhaps in combination with other exotoxins, appears to be responsible for the enhanced pathogenicity of CA-MRSA strains and instrumental in producing necrotic skin lesions and necrotizing pneumonia. Severe invasive disease, such as necrotizing pneumonia and necrotizing fasciitis, appears to be more common with CA-MRSA than methicillin-sensitive *S. aureus* (MSSA) or HA-MRSA. Furthermore, unlike HA-MRSA, which is considered an opportunistic pathogen, CA-MRSA causes infection in healthy, predominantly young hosts who have no predisposing comorbidities.[8]

### Epidemiology

Perhaps the most remarkable feature of the CA-MRSA genotype is its evolutionary success, resulting in its rapid worldwide clonal emergence. The small SCCmec IV allele seems to carry little fitness cost, allowing CA-MRSA to thrive and spread readily outside the hospital environment, unlike HA-MRSA strains which require the hospital milieu for sustained survival. CA-MRSA strains were first reported in the 1980s, and were responsible for an outbreak of infection in intravenous drug users in North America.[5] Later outbreaks were documented in separate community populations such as Australian aborigines, Native Americans, soldiers, male homosexuals, prisoners, and athletes. Although the reasons for MRSA emergence in these groups is not clear, possible commonalities include crowding, poor hygiene, socioeconomic factors, and extensive previous antibiotic use.[6–9] Consistently high prevalence rates are found in the USA, Australia, South America, Japan, and southern Europe, whereas prevalence rates are low in Scandinavia, The Netherlands, and Switzerland.

As in other countries, the prevalence of CA-MRSA has increased in Saudi Arabia in the past 10 years, and CA-MRSA infections in healthy individuals without established risk factors have now been documented in the community in Saudi Arabia. The first CA-MRSA infections were reported from King Fahd Hospital of the university in the Eastern Province[9] and within a short period of time, these strains spread widely in the community. In a study conducted on the west coast of Saudi Arabia, the prevalence of community isolates of MRSA was 15.8% of all MRSA isolates, or 1.1 per 1 000 admissions.[10] Between 1999 and 2003, in Dhahran Medical Center, MRSA constituted 6% of all *S. aureus* isolates; the proportion had increased from 2% in 1999 to 8% in 2003. Of all MRSA isolates, 62% represented community-acquired infection. The proportion of community-acquired isolates increased from 41.7% in 1999 to 66.6% in 2002.[11] Moussa *et al*. studied the genotypes of MRSA from Riyadh and found that CA-MRSA strains harbored the SCCmec type IV element and the PVL genes.[12] The changing epidemiology of *S. aureus* further recorded in another study from King Fahd Hospital of the University revealed that between 2001 and 2008, both the percentage of CA-MRSA isolates as well as the overall number of CA-MRSA isolates increased. By 2008, of all MRSA isolates, 73% represented community-acquired infection and the prevalence of CA-MRSA infections increased from 9.9 per 10 000 admissions in 2001 to 67 per 10 000 admissions in 2008.[13]

Of concern is the recent report of severe cases of invasive infections and sepsis among children caused by CA-MRSA from King Saud Hospital of the university. Between January 2005 and March 2008, five (6%) previously healthy children with invasive CA-MRSA infections were identified from 80 children with community-onset MRSA infections.[14]

### Clinical spectrum of disease

The spectrum of clinical infections caused by CA-MRSA is similar to that caused by MSSA[1,2] but clearly distinct from that caused by HA-MRSA. HA-MRSA commonly causes bloodstream and respiratory tracts infections, whereas CA-MRSA has predominantly been isolated from skin and soft tissue infections (SSTIs).[7] Although CA-MRSA infections are commonly mild, they may also be severe, and can result in hospitalization and subsequent mortality.[12,15]

SSTIs, specifically furuncles, carbuncles, abscesses, and cellulitis, are the most frequently reported clinical manifestations.[1] MRSA skin lesions are frequently confused with spider bites by both patients and clinicians. The severity of CA-MRSA SSTIs varies from mild superficial infections to deeper soft-tissue abscesses, requiring hospital admission for surgical incision and drainage and parenteral antibiotics. Anecdotal reports suggest that recurrent MRSA skin infections and clustering of infections within a household are relatively common.

Head and neck CA-MRSA infections such as cervical lymphadenitis, otitis externa, otitis media with otorrhea, or acute mastoiditis and retropharyngeal abscess are also being encountered with increasing frequency.[16,17] The retropharyngeal abscesses can extend down into the mediastinum and may be associated with jugular vein thrombosis, similar to the classic Lemierre’s syndrome. An increasing number of patients with periorbital and orbital infections as well as other infections related to eye structures caused by CA-MRSA are being encountered in children and adults.[18] Although cases do occur, CA-MRSA is not a common etiology of acute otitis media or acute sinusitis. However, *S. aureus* is an important pathogen to consider in patients with intracranial complications of sinusitis such as epidural or subdural abscesses.[19]

Less commonly, CA-MRSA has been associated with severe and invasive staphylococcal infections in the community, including necrotizing pneumonia and empyema, sepsis syndrome, musculoskeletal infections including pyomyositis and osteomyelitis, necrotizing fasciitis, purpura fulminans, and disseminated infections with septic emboli.[1,12,20] Invasive manifestations occur as complications of preceding SSTIs or viral respiratory tract infections (particularly influenza), as well as in otherwise healthy persons without recognized preceding infections or risk factors.

### Treatment

To avoid clinical complications, clinicians should now consider MRSA as a potential pathogen in patients with suspected *S. aureus* infections in the community. Clinicians should obtain appropriate material for bacterial culture follow-up on the results of susceptibility testing of all *S. aureus* isolates, and recommend surgical drainage of infections when feasible. The primary treatment modality of skin and soft tissue abscesses is incision and drainage. One study demonstrated that with lesions smaller than 5 cm in diameter, outcomes are favorable with incision and drainage alone.[21]

Unlike HA-MRSA, most CA-MRSA isolates are susceptible to several antimicrobial agents. In cases of CA-MRSA infection not requiring hospitalization but for which antibiotics are deemed necessary, oral antibiotics such as TMP-SMX, doxycycline, and clindamycin may be appropriate.[22] The combination of TMP-SMX and rifampicin has also been recommended. In cellulitis patients in whom CA-MRSA is suspected but in whom infection with *Streptococcus pyogenes* cannot be ruled out, some experts do not recommend treating empirically with TMP-SMX or doxycycline monotherapy because of reported streptococcal resistance to these antibiotics. Some researchers suggest a combination of clindamycin with either TMP-SMX or tetracycline.[22] Clindamycin has been used successfully to treat a variety of CA-MRSA infections, including soft tissue infections, pneumonia, and musculoskeletal infections. The possibility of inducible clindamycin resistance has discouraged some physicians from prescribing clindamycin.[23] Strains with inducible resistance will test clindamycin-susceptible *in vitro*, but are erythromycin-resistant. If inducible resistance is present, there is a potential for treatment failure with clindamycin, despite the culture and sensitivity report indicating susceptibility. Antibiotics should be adjusted once culture results are known. Patients treated on an outpatient basis should be clearly instructed to report promptly if they develop systemic symptoms or worsening local symptoms or if their symptoms do not improve within 48 hours.

For patients with invasive infections that may have been caused by CA-MRSA, vancomycin, teicoplanin, tigecycline, and linezolid represent appropriate empirical therapeutic options.[2,21]

### Prevention and control

CA-MRSA outbreaks have been controlled with strategies focusing on wound care and containment, enhanced hygiene, and regular cleaning of frequently touched environmental surfaces. Persons with active infections should be restricted from certain activities such as contact sports and daycare if their wound drainage cannot be contained. Local health authorities should be informed of suspected outbreaks, and additional related cases should also receive adequate treatment. Patient education regarding the nature of the disease, its spread, and the possibility of recurrence is important. Providing antibiotic prophylaxis to family members is currently not recommended, and administering decolonization regimens to whole families has not been studied, but may need to be used in specific circumstances.[24]

## CONCLUSION

CA-MRSA has emerged as an important infection in the community. The rate of CA-MRSA infection is rapidly increasing and has become a threat to the community and to persons with unknown risk factors. Appropriate adjustment of treatment regimens and enforcement of better hygiene practices should prevent further evolution and spread of these highly adapted infectious agents.
